# Transcriptional activation of yeast genes disrupts intragenic nucleosome phasing

**DOI:** 10.1093/nar/gks870

**Published:** 2012-09-24

**Authors:** Feng Cui, Hope A. Cole, David J. Clark, Victor B. Zhurkin

**Affiliations:** ^1^Laboratory of Cell Biology, Center for Cancer Research, National Cancer Institute, Building 37, Room 3035A, Convent Dr., and ^2^Program in Genomics of Differentiation, National Institute for Child Health and Human Development, Building 6A, Room 2A14, 6 Center Dr., National Institutes of Health, 9000 Rockville Pike, Bethesda, MD 20892, USA

## Abstract

Nucleosomes often undergo extensive rearrangement when genes are activated for transcription. We have shown previously, using paired-end sequencing of yeast nucleosomes, that major changes in chromatin structure occur when genes are activated by 3-aminotriazole (3AT), an inducer of the transcriptional activator Gcn4. Here, we provide a global analysis of these data. At the genomic level, nucleosomes are regularly phased relative to the transcription start site. However, for a subset of 234 strongly induced genes, this phasing is much more irregular after induction, consistent with the loss of some nucleosomes and the re-positioning of the remaining nucleosomes. To address the nature of this rearrangement, we developed the inter-nucleosome distance auto-correlation (DAC) function. At long range, DAC analysis indicates that nucleosomes have an average spacing of 162 bp, consistent with the reported repeat length. At short range, DAC reveals a 10.25-bp periodicity, implying that nucleosomes in overlapping positions are rotationally related. DAC analysis of the 3AT-induced genes suggests that transcription activation coincides with rearrangement of nucleosomes into irregular arrays with longer spacing. Sequence analysis of the +1 nucleosomes belonging to the 45 most strongly activated genes reveals a distinctive periodic oscillation in the A/T-dinucleotide occurrence that is present throughout the nucleosome and extends into the linker. This unusual pattern suggests that the +1 nucleosomes might be prone to sliding, thereby facilitating transcription.

## INTRODUCTION

The nucleosomal organization of eukaryotic chromatin is critical for gene regulation (1). The high-resolution X-ray structure of the nucleosome core particle (NCP) (2) provides the detailed information necessary for linking together the primary (or linear, 1D) structure of chromatin (i.e. the nucleosome positions on DNA) and the spatial, 3D, organization of chromatin operative in DNA function. This field has developed intensively during recent years, with emphasis both on the 1D (3) and 3D (4) aspects of the problem. Recent studies of genome-wide nucleosome positioning reveal the presence of a ∼200-bp nucleosome-depleted region (NDR) at the promoters of most genes (5,6). The NDR is typically flanked by phased nucleosomes, which form a highly regular array, extending into the gene body. This stereotypical feature of chromatin is well conserved in different species (7–10). There is also evidence that some nucleosome loss occurs from the coding regions of the most heavily transcribed genes (11–16).

Nucleosome positioning is characterized by two parameters: rotational positioning, defined by the side of the DNA helix that faces the histones, and translational positioning, defined by the nucleosome midpoint (or dyad) with regard to the DNA sequence (17). The structural rules governing rotational nucleosome positioning are believed to be related to the sequence-dependent preferences for DNA deformation, e.g. bending (18). In particular, the A/T-containing dimeric steps AA:TT, AT and TA (denoted as WW) preferentially occur where the DNA is bent into the minor groove, while G/C-containing dimers GG:CC, GC and CG (denoted as SS) are frequently situated at the sites where DNA is bent toward the major groove. The occurrences of WW and SS dimers in nucleosome core DNA both display sinusoidal patterns with ∼10-bp periodicity, but they are ∼5 bp out of phase with one another. These sequence patterns are observed in nucleosomal DNA from chicken (18), yeast (7,8), fruit fly (9), nematode (19) and human (20), indicating that the sequence rules for rotational positioning are essentially the same across species. In contrast, the DNA sequence patterns specifying translational nucleosome positioning are far from clear. The only well-established feature is the tendency of long A/T-rich fragments, and the A-tracts in particular, to be excluded from nucleosomes (21–23). Furthermore, DNA-binding transcription factors and chromatin remodeling enzymes also affect nucleosome positioning (reviewed in 24).

Recently, using genome-wide paired-end sequencing of yeast nucleosomes, we showed that nucleosomes occupy one of several mutually exclusive, overlapping positions, termed a position cluster, which often includes a dominant position (16). Thus, a population of yeast cells exhibits significant heterogeneity in nucleosome positioning. Furthermore, we found that the nucleosome position cluster organization on the coding regions of genes induced by 3-aminotriazole (3AT), an inducer of the transcriptional activator Gcn4 (25), is heavily disrupted (16). Specifically, we observed altered inter-nucleosome spacing at some genes, as well as severe nucleosome loss over the coding region that can extend into neighboring genes (16). In summary, both single-gene and genome-wide studies suggest that extensive nucleosome rearrangements are induced by gene activation. Importantly, the critical details of how nucleosomes shift their positions relative to each other have not yet been analyzed at the genomic level.

Here, we analyze the nucleosomal relocations that occur upon induction of transcription by 3AT at the genomic level, using the DAC function. To eliminate the ‘noise’ in the datasets caused by ‘contamination’ of core DNA by the fragments much longer and much shorter than 147 bp, we focus on the nucleosomal DNA whose lengths are around 147 bp. Our analysis shows that the prevalent distance between nucleosomes is 162 bp, consistent with the nucleosome repeat length (NRL) in yeast, which is 160–165 bp (26). In addition, nucleosome positions are most frequently separated by multiples of 10.25 bp, suggesting that positions belonging to the same cluster are rotationally related. Comparison of the DAC functions for control and 3AT-treated cells reveals no substantial difference at the genome level (∼4700 genes in total). However, detailed analysis of inter-nucleosome distances for ∼250 genes that are strongly induced by 3AT suggests that nucleosome positions change dramatically after 3AT treatment, in some cases resulting in the formation of irregular nucleosomal arrays with altered spacing. Moreover, the +1 nucleosomes on the most heavily induced genes are characterized by an unusual WW pattern, which suggests that these +1 nucleosomes might be more prone to sliding.

## MATERIALS AND METHODS

### Datasets

The paired-end nucleosome sequence data from 3AT-treated cells and control yeast cells (CC) have been described previously (16,27). The paired reads of 40 nt each were aligned to the *Saccharomyces cerevisiae* genome using ELAND (Illumina). Only the reads uniquely aligned to the genome with no mismatch were selected.

For both the control and 3AT datasets, the length of nucleosomal DNA ranges from ∼120 to 180 bp (Supplementary Figure S1). The highest peak in both distributions occurs in the interval from 147 to 152 bp. Therefore, all DNA fragments of 147–152 bp were selected for further analysis (5 368 041 and 3 853 385 sequences from the CC and 3AT sets, respectively). Note that the length of DNA in the highest resolution crystal structure of the NCP is 147 bp (2). For brevity, we denote the selected DNA sequences as NCP sequences. Nucleosome sequences originating from the highly repeated ribosomal DNA locus (chromosome XII: 451 419–468 930 bp) were excluded from the analysis.

For a given gene, the +1 nucleosome is defined by NCP sequences that have midpoints located between coordinate +1 (i.e. the transcription start site, TSS) and coordinate +140 of the gene. The sequences were oriented according to the direction of transcription.

### Inter-nucleosome distance correlation functions

To describe relative positions of nucleosomes, we used the distance auto-correlation (DAC) function (Supplementary Figure S2). For each pair of NCP sequences, the distance between the NCP start locations was calculated. Then the occurrences of all distances are summed up for both strands. Note that multiple occurrences of nucleosomes in the same position were counted multiple times—that is, if two specific nucleosome sequences occur 5 and 10 times, respectively, the corresponding auto-correlation will be calculated as 5 × 10 = 50. In this regard, our DAC differs from the ‘coincidence number’ as a function of ‘start-to-start distance’ used by Valouev *et al.* (28), who counted multiple occurrences of nucleosomes in the same position only once. We also calculated the ‘center-to-center’ distances; the results were nearly identical to those obtained using the ‘start-to-start’ procedure.

To compare the nucleosome positions in two different datasets (CC and 3AT, for instance), we used the distance cross-correlation (DCC) function. It is similar to the auto-correlation function, except that the distances were calculated between the start location of each nucleosome in one dataset and all of the start locations of nucleosomes in the other dataset.

## RESULTS

### Nucleosomes are regularly spaced and adopt rotationally related positions

**Figure 1.**
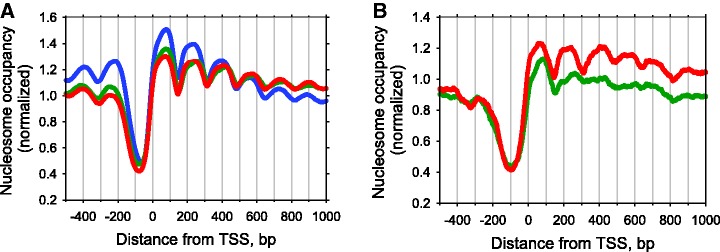
Nucleosome organization around the 5′-end of yeast genes. (**A**) Overlays of nucleosome occupancy profiles of 4792 *S. cerevisiae* genes (30) relative to TSS (position 0). Nucleosome occupancy values are either taken directly from Kaplan *et al.* (29) (blue) or recalculated from Cole *et al. (*16,27): 3AT set (green) and CC set (red), respectively. In the latter two cases, the NCP fragments 147–152 bp in length were selected to calculate occupancy profiles. (**B**) Nucleosome occupancy map for 234 genes (out of 4792 genes) that are induced by 3AT by more than 2-fold (25). Note that the occupancy value at each nucleotide is normalized by summing all the nucleosome sequences covering this nucleotide and dividing that number by the average number of nucleosome sequences per base pair across the genome.

The CC and 3AT datasets contain ∼5.4 and 3.9 million DNA fragments of core particle length (147–152 bp), respectively. These NCP sequences were used to build nucleosome maps centered on the 5′-ends (TSSs) of all yeast genes. Both the CC and 3AT nucleosome maps are similar to the *in vivo* map (29) obtained for yeast grown on the yeast extract-peptone-dextrose medium (YPD), with a Pearson’s correlation coefficient of 0.56 (*P* < 10^−^^15^) between the CC map and the YPD map (Supplementary Figure S3A). Comparison of the nucleosome organization around the 5′-end of genes (Figure 1A) reveals a well-established pattern (7,8): nucleosomes are depleted upstream of the TSS and are strongly ordered in the gene body, with the distances between the peaks varying between 160 and 170 bp. As noted earlier (30), the+1 nucleosomes are especially well positioned and form the strongest peak in the nucleosome occupancy profile (Figure 1A).

**Figure 2.**
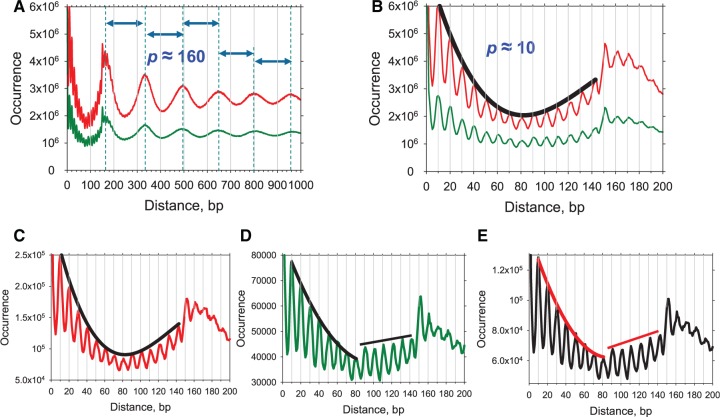
DAC function for nucleosomes in the control (red) and 3AT-treated (green) cells. (**A** and **B**) General and detailed views of the function for all NCP sequences 147–152 bp in length. The positions of the peaks in A are denoted by dashed lines, separated by ∼160 bp. A continuous envelope curve in B passes through the peaks of the profile for the control set. The envelope curve for the 3AT set is omitted for clarity. (**C** and **D**) Detailed views of the auto-correlation function for NCP sequences from 234 genes induced by 3AT (±200 bp). The envelope curve is continuous for the control set (C), but not for the 3AT set (D), in which a sharp increase in the profile of the auto-correlation function occurs at 80–90 bp. (**E**) Detailed view of the cross-correlation function for nucleosomes in the control and 3AT sets (see ‘Methods’ section). Envelope curves are shown as in D.

**Figure 3.**
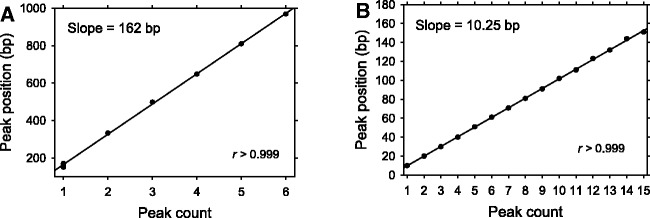
Linear regression analysis of the peak positions for the inter-nucleosome DAC function (Figure 2). (**A**) Linear fit of the peaks shown in Figure 2A (positions 151, 162, 171, 333, 498, 648, 810 and 969). The straight line has a slope of 162 bp (*r* = 0.9999). (**B**) Linear approximation of the sub-peaks shown in Figure 2B (positions 10, 20, 30, 40, 51, 61, 71, 81, 91, 102, 111, 123, 132 and 144 bp). The straight line has a slope of 10.25 bp (*r* = 0.9999).

We further examined regularity in nucleosome positions on the genome scale by calculating the DAC function (see ‘Methods’ section and Supplementary Figure S2). This function exhibits a series of well-defined peaks separated by 150–170 bp (Figure 2A). The first peak is located at ∼160 bp and is split into three sub-peaks at 151, 162 and 171 bp (Figure 2B), whereas the other peaks, located between 300 and 1000 bp, each have a single maximum. The peak coordinates are approximated by a straight line with a slope of 162 bp (Figure 3A). This value can be used as a measure of ‘average’ genome-wide periodicity in the nucleosome positions. Remarkably, this period is practically identical to the NRL in yeast, 162–165 bp (26,31,32).

Note that the two periods mentioned above have essentially different meanings (see Supplementary Figure S2 for an illustration). The NRL is determined from the sizes of the oligo-nucleosomal DNA fragments in the ‘ladder’ observed after separation in an agarose gel. Each oligo-nucleosomal band represents a population of chromatin fragments with a certain number of nucleosomes on the same DNA molecule, which cannot overlap (Supplementary Figure S2A). The NRL that is inferred from these bands therefore describes the relationship between physically connected nucleosomes. In contrast, the DAC function (Figure 2A) was used here to analyze the mono-nucleosome population, which are no longer physically linked to one another (Supplementary Figure S2B). Therefore the DAC function describes the distance relationship between all nucleosomes, not just those on the same DNA molecule. That the two repeat lengths are so close indicates that yeast nucleosome positioning is characterized by a strong long-range order which is systematically reproduced in all cells, genome-wide.

A closer inspection of the DAC function reveals a very clear fine structure, with sub-peaks at 10, 20, 30, 40, 51, 61 bp, etc. (Figure 2B). The ∼10-bp periodicity suggests that nucleosome positions tend to overlap each other by a multiple of 10 bp, and therefore that they are rotationally related. That is, the DNA is wrapped in nucleosomes in such a way that the orientation of the helix with respect to the histone surface is the same in these overlapping positions. Interestingly, the high-resolution profiles presented in Figure 2B allow one to measure the locations of the sub-peaks unambiguously and to distinguish, for example, between 50 and 51 bp. Based on these measurements, we conclude that the periodicity of DNA secondary structure is higher than 10.0 bp per turn. The linear regression analysis of the sub-peak positions renders a periodicity of 10.25 bp (Figure 3B), which is very close to the value of 10.18 ± 0.05 bp per turn, obtained by Hayes *et al.* (33) who measured average helical periodicity of 5S DNA in a reconstituted nucleosome using hydroxyl radical footprinting. This value is also close to the ‘local frame twist’ of 10.30 bp per turn in the 147-bp NCP crystal structure (2), and of 10.23 and 10.15 bp per turn in the two 146-bp NCP structures (34).

The sub-peaks we observed at 10, 20, 30 bp, etc. (Figure 2B) appear to be smoother and more regular than those in the start-to-start distance profiles published for nematode nucleosomes (28). There are several alternative explanations for this difference. First, the sizes of the nematode nucleosomal sequences were not determined, because only one end of each fragment was sequenced and aligned to the genome. That is, the nematode data can be directly compared to the total set of yeast nucleosomes with the length ranging from ∼120 to 180 bp (Supplementary Figure S4), but not with the set of NCP sequences having rigorously defined lengths of 147–152 bp (Figure 2B). However, even if the two total sets are considered, the yeast profiles reveal stronger periodicities in inter-nucleosome distances compared to nematode. (Note that this tendency holds both for the local period of ∼10 bp and for the long-range chromatin structure period of ∼160–170 bp, see Supplementary Figure S4.) A second explanation implies that indeed, the yeast nucleosomes are in general more regularly positioned than in nematode. This is a reasonable possibility, given that *C. elegans* has many cell types and thus, its chromatin organization is likely to be more heterogeneous than in yeast. To clarify this issue, the lengths of the nematode nucleosomal DNA sequences are needed.

### Activation disrupts nucleosome clusters: a case study of *PET56–HIS3* chromatin

**Figure 4.**
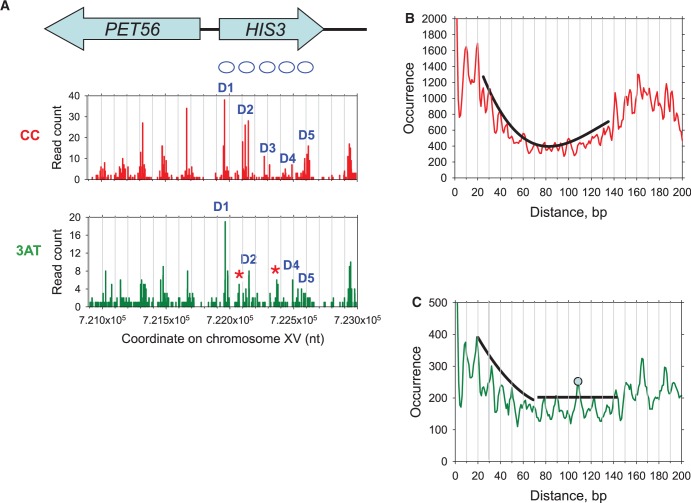
Nucleosome position clusters and distance correlation function for nucleosomes at the *PET56–HIS3* locus. Only NCP sequences from this locus that were 147–152 bp in length were used for the analyses (between coordinates 721 000 and 723 000 on chromosome XV). (**A**) Nucleosome position clusters for the CC and 3AT sets. D1–D5: dominant positions adopted by nucleosomes on *HIS3* (35). The asterisks indicate new clusters formed after 3AT induction. The numbers of sequences at each dyad position are plotted. The dyad was defined by the coordinate of the central nucleotide in each sequence; if this number was non-integral, it was rounded up. (**B** and **C**) Distance correlation function for the nucleosomes in the CC and 3AT sets, respectively. The envelope curves are shown in black. The prominent sub-peak at ∼110 bp (C) corresponds to the distance between nucleosome position D1 and the 3AT-induced position [the left asterisk in A, lower panel].

Recently, we demonstrated for selected genes that nucleosome positions are naturally divided into groups which we defined as ‘position clusters’ (16). Each position cluster is usually composed of a dominant position and several alternatives. Activation by 3AT, results in a major disruption of nucleosome position clusters, sometimes with altered spacing, in the coding regions of dozens of Gcn4-dependent genes (16). On *HIS3*, for example, 3AT induction results in the disruption of two nucleosome position clusters (D3 and D4) (35) and the appearance of two new clusters (denoted by asterisks in Figure 4A, lower panel). These changes in the chromatin structure apparently alter the pattern of nucleosome spacing on *HIS3*.

To analyze the changes in the *PET56–HIS3* chromatin rigorously, we calculated the DAC function for nucleosome sequences in the CC and 3AT sets (Figure 4B and C). We found that strong sub-peaks at 10 and 20 bp are observed in both profiles, indicating that nucleosome positions within the clusters are rotationally related. Naturally, these profiles are not as regular as in Figure 2A, because of a limited statistics. The main difference between the two correlation functions lies in the interval from 80 to 140 bp. For the CC set, the profile is quite smooth with a continuous envelope curve (Figure 4B), similar to that for all nucleosome positions genome-wide (Figure 2B). This indicates that the nucleosomes are positioned in a regular array of clusters. In contrast, the profile for the 3AT set cannot be fit with a single envelope curve—the general pattern of the local sub-peaks at distances 80 bp and more is rather irregular (Figure 4C) and clearly differs from the smooth monotonic pattern of sub-peaks between 20 and 70 bp observed for the CC set (Figure 4B). In particular, the sub-peak at ∼110 bp is much higher than its neighbors, forming a local maximum. Detailed analysis showed that this prominent sub-peak corresponds to the distance between the major nucleosome position D1 and the position with a relatively high occupancy in the 3AT set, which was strongly under-represented in the CC set (the left asterisk in Figure 4A, lower panel). Thus, we conclude that the changes in nucleosome positions resulting from 3AT activation can be detected by the distance correlation function, at least in the case of *HIS3*.

### Local changes in nucleosome organization upon 3AT induction

To determine whether similar nucleosome rearrangements occur on other 3AT-induced genes, we selected 234 genes (including *HIS3*) that are induced >2-fold (25) and repeated our analyses on this subset of genes. Note that the length distribution of nucleosomal DNA sequences in these 234 genes is nearly identical to the distribution observed genome-wide; this holds both for the 3AT and CC sets (Supplementary Figure S1). The regular nucleosome pattern downstream of the 5′-end of these genes was clearly disrupted in the 3AT set compared to the CC set (Figure 1B), consistent with our earlier observations for selected genes (16). In particular, all the intragenic positions are partially depleted compared to the genome-wide distribution (Figure 1B). This effect is relatively small for the+1 position, but for the positions further downstream of the NDR (+2, +3, etc.) the nucleosome occupancy gradually decreases and becomes less than the genome-wide level.

The DAC function was computed for nucleosome positions on these genes (within ±200 bp from the gene body). For the CC set, the profile of the function gradually decreases in the interval from 10 to 80 bp and then increases in the interval from 90 to 140 bp, so that a continuous envelope curve can be drawn through the peaks of the function (Figure 2C). In the case of the 3AT set, the downward envelope curve in the interval from 10 to 80 bp resembles that in the control set (Figure 2D). However, at distances of 90 bp and more, the peaks of the auto-correlation function are markedly higher, as observed for *PET56–HIS3* chromatin (Figure 4C). The straight envelope line observed in the interval from 90 to 140 bp (Figure 2D) is qualitatively different from the smooth envelope curve obtained for the CC set (Figure 2C).

**Figure 5.**
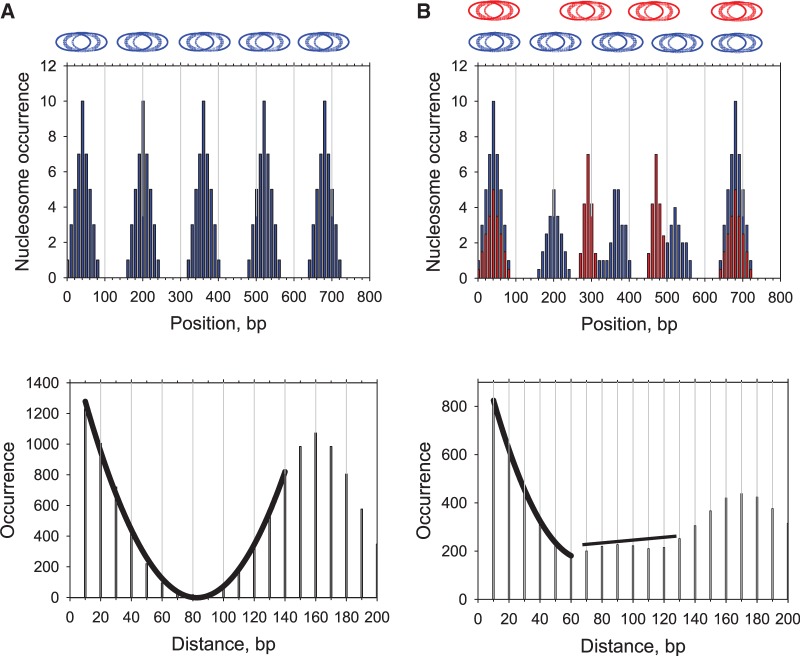
Representative examples of the DAC function for cells in which nucleosomes are present in arrays with ‘normal’ spacing (160 bp) (**A**) and for a mixed population in which some cells have nucleosomes with 160 bp spacing on the gene (blue), and other cells have nucleosomes in arrays with longer spacing (180–250 bp in this example, shown in red) (**B**). The nucleosome occurrences at each nucleotide are presented as histograms. The corresponding DAC functions are plotted below, with the envelope curves passing through the peaks of the occurrence profiles.

The most likely interpretation of the discontinuity observed at a distance of 90 bp in the 3AT set (Figure 2D) is that overlapping positions separated by 90 bp or greater are more frequent in the 3AT set, compared to the CC set. To determine whether the discontinuity at 90 bp can be accounted for in this way, we analyzed two hypothetical cases. To model the CC data, we argued that the 3AT-induced genes are organized into nucleosomal arrays with the same spacing as genomic chromatin (∼160 bp). To model the 3AT data, we postulated that the 3AT-induced genes are organized either in arrays the same as those in control cells (160 bp spacing), or into irregular arrays with longer spacing, varying from 180 to 250 bp (Figure 5). The DAC function for arrays with 160 bp spacing displays a fine structure similar to that observed for the CC set, with a continuous envelope curve (compare Figures 5A and 2C). The DAC function for overlapping arrays with different spacings resembles that observed for the 3AT set, predicting the complex envelope curve quite well (compare Figures 5B and 2D). This simple test suggests that nucleosomes in control cells can be modeled by a set of arrays with uniform spacing, as shown in Figure 5A. In 3AT-treated cells, however, nucleosomes on 3AT-activated genes are likely to be organized either in arrays with the spacing characteristic of the genome as a whole (∼160 bp), as in CC cells, or alternatively, in irregular arrays with longer spacing and therefore fewer nucleosomes, as shown in Figure 5B.

To compare nucleosome positions directly (on the 234 genes induced by 3AT) in the CC and 3AT sets, we calculated the cross-correlation function (see ‘Methods’ section). This function has the highest value at position 0 (beyond the scale of Figure 2E), indicating that most of the nucleosomes in the two sets share the same positions. In addition, the cross-correlation function exhibits a discontinuity at 90 bp, similar to that observed for the 3AT set (Figure 2D). This observation suggests that at least some nucleosomes in control cells shifted position significantly in 3AT-induced cells.

Overall, the genome-wide CC and 3AT nucleosome occupancy maps are in excellent agreement, with a correlation of 0.96 (Supplementary Figure S3B). Also, the fine structures of the two DAC functions look very similar in terms of the overall trend displayed by their profiles (Figure 2B). In particular, continuous envelope curves can be drawn on top of the sub-peaks for both functions indicating that both the CC and 3AT sets reveal the same (or very similar) general patterns of inter-nucleosome spacing genome-wide. Thus, we conclude that upon 3AT activation, the nucleosome organization remains globally unchanged but alters noticeably on the 3AT-induced genes (25).

### Insignificant impact of MNase cleavage bias on nucleosome sequence patterns

Next, we assessed the impact of MNase cleavage bias on the DNA sequence patterns observed in yeast nucleosomes. It is well documented that MNase cuts predominantly at A/T sequences, in both free DNA (36,37) and in the linker DNA between nucleosomes (38–40). Consistent with these observations, we found that the consensus MNase cleavage sequence is WWT|TG, where the vertical bar denotes the cutting position and W stands for A or T (Supplementary Table S1). Note that at the two positions denoted T|T above, thymines occur in ∼50% of all sequences, whereas occurrence of W (either A or T) at these positions is as high as 83 and 93% (see positions 0 and +1 in Supplementary Table S1). However, this consensus is very weak because it describes <5% of the actual cleavage sites. A much more predictive consensus sequence is W|W, which accounts for ∼80% of cleavage sites, and agrees with that reported by Field *et al.* (39). Taking into account the fact that the yeast genome is ∼60% AT, the WW dimers should occur once every 3 bp on average. Thus, predicted cleavage sites are so common in yeast DNA that the MNase cleavage bias is likely to be practically unimportant. Nevertheless, GC-rich regions may present local problems.

**Figure 6.**
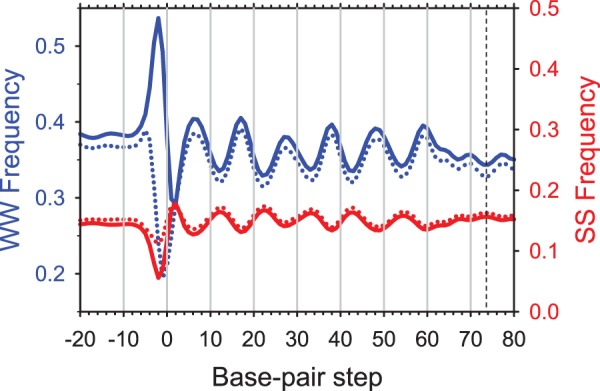
Occurrences of the WW (AA:TT + AT + TA) and SS (GG:CC + GC +CG) dimers in the NCP sequences (L = 147–152 bp; CC set). Solid lines: The combined frequencies of occurrence of WW (blue) and SS (red) dimers for all NCP sequences. Dotted lines: The same for the subset of NCP sequences (343 794 or ∼7% of the total) with no WW dimers at the terminal positions 0 and +1. Three base pair running averages are shown, symmetrized with respect to the dyad at base-pair step 73.5 (denoted by a dashed line). The NCP sequences are ‘center-aligned’ with 20-bp extension in both directions.

To determine whether MNase cleavage bias introduced a significant bias in the DNA sequence patterns traditionally associated with nucleosome positioning (18), we compared the NCP sequences with non-preferred MNase cutting sites at the ends with the total NCP set. We selected the NCP fragments containing no A or T at positions 0 and +1 and calculated the frequencies of occurrence for WW and SS dimers (Figure 6). As expected, the WW profile for this subset displays a dip close to the ends of the NCP fragments (around position 0 in Figure 6). In contrast, the WW profile for the whole dataset shows a peak at the corresponding position, reflecting the abundance of A and T at the NCP ends (Supplementary Table S1). Both datasets exhibit the well-known sinusoidal WW and SS patterns with ∼10-bp periodicity and shifted from each other by ∼5 bp (Figure 6). Remarkably, the two curves agree very well within the NCP region indicating that the DNA fragments with and without preferential MNase cutting sites at the ends have practically the same WW and SS profiles, which are characteristic of positioned nucleosomes (reviewed in 41).

It might be expected that the periodic WW pattern internal to the nucleosome represents a series of enhanced cleavage sites for MNase, since the enzyme prefers to cut at WW (36,37). However, the nucleosomal WW motifs are located where the minor groove faces in, and MNase prefers to cut DNA where the minor groove faces out. Consequently, it is unlikely that the periodic WW motifs are preferred cleavage sites for MNase. On the other hand, those WW dinucleotides that are located where the minor groove faces out (the troughs in the WW profile; Figure 6) might be preferred cleavage sites for MNase.

Based on this analysis, we suggest that the sequence preference of MNase cleavage has little impact (if any) on the observed periodic sequence patterns generally believed to guide nucleosome positioning, at least at the level of dimeric steps. A recent study of nucleosomes reconstituted on genomic DNA reached the same conclusion with regard to MNase bias (42).

### DNA sequence patterns in +1 nucleosomes

**Figure 7.**
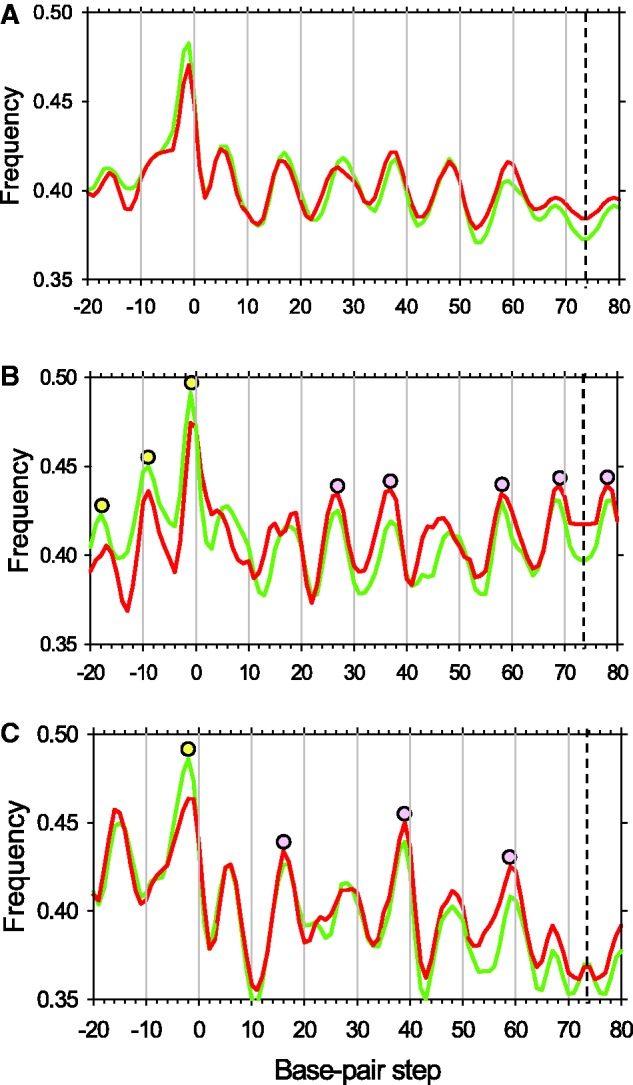
WW profiles in +1 nucleosomes for the 234 genes induced by 3AT: all 234 genes (**A**), the 45 most strongly induced (top-45) genes (**B**) and the 45 least induced (bottom-45) genes (**C**). Three base pair running averages are shown, symmetrized with respect to the dyad at base-pair step 73.5 (denoted by a dashed line). The NCP sequences are ‘center-aligned’ with 20-bp extension in both directions. The curves for the CC and 3AT sets are depicted in red and green, respectively. The pronounced peaks are denoted by filled circles; those within the core DNA are shown in purple, while those located in linker DNA are shown in yellow.

The highly organized chromatin structure around the TSS *in vivo* has been described in many studies (5–10). A common feature is the presence of a nucleosome-depleted promoter region flanked by well-positioned −1 and +1 nucleosomes. The downstream +1 nucleosome is critically situated for playing a role in regulating transcription initiation and elongation. We observed that, after activation of transcription, the +1 nucleosome positions are shifted slightly (but noticeably) downstream on the subset of 3AT-activated genes (Figure 1B). Therefore, we analyzed in detail the sequence organization of these nucleosomes. Overall, the +1 nucleosomes are characterized by a WW profile very similar to that observed for all nucleosomes genome-wide (compare Figures 6 and 7A). Significantly different patterns were observed, however, when we compared the most induced genes with the least induced genes within the set of 234 genes activated by 3AT (the top and bottom 20% of genes; 45 genes in each group).

Nucleosomes from the top-45 group have a WW profile with numerous strong peaks separated by intervals of ∼10 bp (Figure 7B). Importantly, the strong WW peaks are distributed throughout the nucleosome, including the linkers and the central part of the core. The latter is unusual because typically, the center of the nucleosomes is more G/C-rich than the termini, and the central WW peaks are much less pronounced, both *in vivo* (20,39,43) and *in vitro* (44); see also Figure 6. The G/C-rich center of the nucleosome is thought to serve as a positioning signal (20,39,43). The pseudo-uniform distribution of the WW peaks observed for the top-45 genes (Figure 7B) implies that displacement of nucleosomes by multiples of 10 bp would not result in substantial changes in the energetic cost of DNA deformation. We predict that these +1 nucleosomes are ‘positionally unstable’ (30), and that their relocation during transcription would occur relatively easily.

In contrast, the +1 nucleosomes from the bottom-45 group have a WW profile with a smaller number of strong peaks separated by longer intervals (Figure 7C). The locations of these peaks (sites #16, 38 and 58) correspond to strong kinks in the nucleosome crystal structures (2,45), where DNA is severely bent into the minor groove. Importantly, A/T-rich sequences are more preferable at these locations than G/C-rich ones, mostly due to two factors. The first is the intrinsic anisotropy of DNA bending; AT-rich DNA bends relatively easily into the minor groove (46,47). The second is electrostatic interactions with the histone arginines that penetrate the minor groove (48,49), which is also more favorable for A/T-rich sequences (48). Therefore, we predict from the WW profile presented in Figure 7C that these +1 nucleosomes will exhibit significant ‘positional stability’ (30).

Moreover, the WW frequency attains its widest minimum in the central part of the nucleosome (Figure 7C); according to the empirical observations mentioned above (20,43), this further increases the predicted stability (7) of the +1 nucleosomes in the bottom-45 group. Finally, note that the major WW peaks are separated now by intervals of ∼20 bp (Figure 7C) rather than ∼10 bp in the previous case (Figure 7B). In other words, the distances between the optimal nucleosome positions have increased from ∼10 to 20 bp, which might be expected to hinder nucleosome relocation.

In addition, we analyzed WW profiles that are not symmetrized with respect to the dyad axis and found that for the 234 genes activated by 3AT, the periodic peaks extend into the downstream linker (Supplementary Figure S5A). Interestingly, this effect is rather strong for the top-45 set (Supplementary Figure S5B), but not for the bottom-45 set (Supplementary Figure S5C). Thus, we predict that the +1 nucleosomes in the top-45 set should slide downstream more easily than their counterparts in the bottom-45 set. All these factors taken together suggest that the +1 nucleosomes in the bottom-45 group will be less easily shifted, which correlates with the weaker induction of these genes.

In summary, we see that there is a clear negative correlation between the 3AT-induced activation of gene transcription and the strength of +1 nucleosome positioning, as deduced from the periodic patterns of WW peaks. This correlation is not found in the −1 and +2 nucleosomes (Supplementary Figure S6), indicating that the unusual DNA sequence patterns described above exist only in +1 nucleosomes.

## DISCUSSION

### A sensitive computational approach to capture local changes in chromatin structure

Revolutionary high-throughput sequencing techniques provide a unique opportunity to generate millions of nucleosomal DNA sequences across a eukaryotic genome (3,50). Importantly, the nucleosomes are not uniformly distributed throughout the genome. Rather, at some locations (e.g. in promoter regions) nucleosomes are depleted (5,6), while at other locations nucleosomes are strongly positioned. Thus, the inter-nucleosome distances can be calculated differently, depending on whether ‘strong’ positions, corresponding to multiple occurrences of nucleosomes, are treated the same as ‘weak’ positions with a single occurrence. That is, should positions with very different occurrences be treated in the same way?

Valouev *et al.* (28) introduced the ‘coincidence number’ in which the strong positions are considered equivalent to the weak ones in the sense that each position is counted once. As a result, the variation of the ‘coincidence number’ is relatively small: the peak-to-trough ratio is 1.33/1.2 (=1.11) (see the red curve in Supplementary Figure S4D). In this study, we have introduced the DAC function, which takes into account the frequency of occurrence of nucleosomes at a given position. Accordingly, the variation of our DAC value is larger: the peak-to-trough ratio is 3.25/1.5 (=2.17) (see the red curve in Supplementary Figure S4B).

As an illustration, we applied our function to the well-studied *HIS3* chromatin, which undergoes substantial changes upon 3AT induction (16,35). In particular, the under-represented position between the D1 and D2 positions in control cells becomes more frequent in 3AT-treated cells (denoted by left asterisk in Figure 4A, lower panel). The distance between this new position (5 occurrences) and D1 (19 occurrences) is 109 bp. This rearrangement of nucleosomes is successfully captured by the DAC function: the occurrence of this particular distance, 109 bp, is increased by 95 (= 5 × 19) and the peak at 109 bp stands out from its neighbors (Figure 4C). In contrast, if each position were counted only once as suggested by Valouev *et al.* (28), the occurrence of this distance would increase by 1 (= 1 × 1), and the peak at 109 bp would be indistinguishable from its neighbors. This example demonstrates that our approach is sensitive enough to detect local changes in nucleosome positioning.

Kaplan *et al.* (29) found that *in vivo* nucleosome maps derived from yeast cells grown in different carbon sources are highly similar to each other. These data imply that the overall chromatin structure remains unchanged when yeast cells are grown in different conditions. It should be noted, however, that these studies (12,29) were based on measurements of nucleosome occupancy. That is, instead of considering the positions of individual nucleosomes, the nucleosome occurrence was averaged over a ∼150 bp interval.

Here, we analyzed the exact position of each nucleosome and found that the DAC functions for nucleosomes genome-wide agree very well for cells with or without 3AT treatment (Figure 2A and B). In contrast, noticeable changes in the inter-nucleosomal distances are observed for the 234 genes strongly induced by 3AT (Figure 2C and D). Our interpretation is that the nucleosome rearrangements occurring upon activation of transcription lead to formation of new nucleosome position clusters and arrays, by analogy with the *HIS3* gene (Figures 4 and 5). In addition to re-positioning of canonical nucleosomes, many of these 234 genes exhibit a major loss of nucleosome occupancy across the entire body of the gene when activated by 3AT (16). This loss of occupancy may reflect activation-induced removal of nucleosomes from the gene (51–53). Alternatively, the apparent loss of occupancy may instead reflect the presence of remodeled nucleosomes on the gene (54), if such nucleosomes are digested more easily by MNase than canonical nucleosomes, and therefore disappear from the maps. If so, this remodeling could be due to ATP-dependent chromatin remodeling activities, such as the RSC (remodels structure of chromatin) complex (55).

In conclusion, our novel method captures the re-positioning of individual nucleosomes upon the activation of several hundred Gcn4-regulated genes and provides critical insight into the nucleosome dynamics that occur when genes are activated, at a global level.

### Advantage of the paired-end nucleosome sequencing method

Until recently, genome-wide maps of nucleosome positions were obtained with the single-end method of sequencing (7,8,10,19,20,28–30), in which only one end of the nucleosomal DNA is sequenced. The location of the other end is not determined experimentally and must be inferred, usually by assuming that it is 147 bp away from the sequenced end. The recent advent of the paired-end sequencing technique made it possible to obtain millions of nucleosomal DNA sequences together with their lengths (16,27,56,57). For yeast chromatin, we can make a direct comparison between results obtained by single-end (7,8,29,30) and by paired-end (16,57) sequencing of nucleosomes.

To evaluate the precision of nucleosome positioning, we calculated the WW periodicity (7–9,18–20) along the core DNA sequences (Figure 6). The ∼55 000 single-end nucleosome positions provided by Mavrich *et al.* (Supplementary Table S1 in Ref. 30) rendered no periodic pattern in the WW distribution (Supplementary Figure S7A). The WW periodicity was detected only after we re-aligned the nucleosome sequences using the WW, SS and other sequence patterns critical for nucleosome positioning (44, ‘Set 2’), see Supplementary Figure S7B. In contrast, the strong periodic WW pattern is clearly visible in the raw data from Cole *et al.* (16); re-alignment did not improve the WW oscillation significantly (Supplementary Figures S7C and D). We obtained a similar result for the nucleosome positions reported by Henikoff and co-authors (57) who also used the paired-end technique (data not shown). Thus, we conclude that the most reliable information on the sequence patterns in positioned nucleosomes is obtained by paired-end sequencing (16,57) without any additional computer alignment.

### Unusual sequence patterns encoded in +1 nucleosomes

One of the characteristic features of nucleosome organization across a eukaryotic genome *in vivo* is that the +1 nucleosome is well-positioned downstream of a NDR. In *Drosophila*, for example, RNA polymerase II pauses as it contacts the +1 nucleosome (9,58,59). Thus, it is believed that +1 nucleosomes are critical for initiation and elongation by the RNA polymerase II transcriptional machinery (60).

The +1 nucleosomes are much less well phased with respect to the TSS in *in vitro* maps, in which nucleosomes are reconstituted by salt gradient dialysis (29,61), suggesting that intrinsic histone–DNA interactions are not the only determinants of +1 nucleosome positioning. It was shown recently that the ATP-dependent chromatin remodeling factors play an important role in maintaining the translational positioning of +1 nucleosomes as well as other nucleosomes downstream (62,63).

Our analysis reveals a clear-cut WW pattern with ∼10-bp periodicity in +1 nucleosomes (Figure 7A), suggesting that these nucleosomes adopt rotationally related positions. In addition, we observed very interesting sequence patterns in nucleosomes on the genes most strongly induced by 3AT (top-45 genes). That is, WW dinucleotides oscillate strongly throughout the entire nucleosome, including the dyad region, and continuing into the linkers (Figure 7B). Moreover, the WW peaks in the linkers are distributed asymmetrically, the linker DNA downstream of the +1 nucleosome having stronger peaks than the upstream linker (Supplementary Figure S5). Apparently, these +1 nucleosomes might easily slide by ∼10 or 20 bp (particularly downstream from the TSS) and still retain the same orientation of the DNA helix relative to the histone core. Importantly, this pattern exists in the +1 nucleosomes before activation (see red curve in Figure 7B), implying that these nucleosomes are prone to re-position, which may facilitate active transcription of the corresponding genes.

This observation is consistent with the pronounced widening of the occupancy peak for the +1 nucleosomes in the top-45 genes (Supplementary Figure S8A). Here, the +1 nucleosomes are distributed so broadly that there is no gap between nucleosomes +1 and +2. In the bottom-45 genes, on the contrary, the peaks for nucleosomes +1 and +2 are clearly separated from each other by a very deep and wide gap, both in 3AT-activated and control cells (Supplementary Figure S8B). Apparently, these +1 nucleosomes are well positioned, in accord with the unusually strong WW rotational positioning signal revealed in this case (Figure 7C). To our knowledge, this is the first evidence that the DNA sequence patterns in +1 nucleosomes might contribute to gene transcription.

Finally, we wish to emphasize the major findings of this study.

- Gene activation results in reduced nucleosome density, which reflects both nucleosome depletion or remodeling and increased inter-nucleosome distances, testifying to the presence of at least two alternative settings of nucleosomes on the induced genes. Thus, 3AT-induced genes undergo major changes in chromatin structure which might facilitate transcription because there are fewer nucleosomes to delay RNA polymerase II and the chromatin is likely to be less compact.
- The +1 nucleosomes on the 45 most strongly activated of these genes reveal an unusual profile for the positioning WW dimers, implying that these nucleosomes are prone to slide upon induction of transcription.

Thus, the inter-nucleosome distance correlation function introduced here can be used at the genome level to detect rearrangement of nucleosomes on highly induced genes, particularly relative to their promoters.

## SUPPLEMENTARY DATA

Supplementary Data are available at NAR Online: Supplementary Table 1 and Supplementary Figures 1–8.

## FUNDING

The Intramural Research Program of the National Institutes of Health (Center for Cancer Research, National Cancer Institute; Program in Genomics of Differentiation, National Institute for Child Health and Human Development). Funding for open access charge: The Intramural Research Program of the National Institutes of Health.

*Conflict of interest statement*. None declared.

## Supplementary Material

Supplementary Data
