# Supplementary material for: Transcriptional activation of yeast genes disrupts intragenic nucleosome phasing
Source: Nucleic Acids Res. 2012 Sep 24;40(21):10753–64. doi: 10.1093/nar/gks870 (PMC3510488; doi:10.1093/nar/gks870)
Supplement: Supplementary Data [file supp_40_21_10753__index.html]

Transcriptional activation of yeast genes disrupts intragenic nucleosome phasing — Transcriptional activation of yeast genes disrupts intragenic nucleosome phasing — Supplementary Data 

# Transcriptional activation of yeast genes disrupts intragenic nucleosome phasing

## Supplementary Data

files

**Files in this Data Supplement:**

- Supplementary Data - pdf file
