# Supplementary material for: Transcriptional activation of yeast genes disrupts intragenic nucleosome phasing
Source: Nucleic Acids Res. 2012 Sep 24;40(21):10753–64. doi: 10.1093/nar/gks870 (PMC3510488; doi:10.1093/nar/gks870)
Supplement: Supplementary Data [file supp_gks870_nar-01482-m-2012-File003.pdf]

Transcriptional activation of yeast genes disrupts intragenic nucleosome phasing

Feng Cui, Hope A. Cole, David J. Clark and Victor B. Zhurkin

Supplementary Material

Figure S1. Length distribution of nucleosomal DNA sequences in two data sets used in this study, the CC set (**A**) and the 3AT set (**B**) (adapted from Cole *et al.* (16,27)). Red and green vertical bars represent genome-wide data; cyan and pink curves are for 234 genes induced by 3AT (± 200 bp). The fraction of sequences of a given length is shown as the percentage of total reads. Vertical lines and arrows represent the DNA fragments treated as NCP sequences.

Figure S2. Distance auto-correlation function for nucleosomes in a single array (**A**) and in multiple overlapping arrays (**B**). In (**B**), the dominant array is denoted D, and the alternative arrays are denoted A–20, A–10, A+10 and A+20. In this schematic representation, nucleosomes in one array are shifted from nucleosomes in the other array by multiples of 10 bp. Arcs represent the "start-to-start" distances between nucleosomes on one strand (top). The occurrences of these distances are averaged with the corresponding occurrences on the complementary strand. The resulting occurrences of distances are plotted at the bottom. Note that in (**B**) the inter-nucleosome distances constitute groups (or clusters) of values separated by multiples of 10 bp.

The scheme in (**A**) corresponds to traditional evaluation of the NRL from the sizes of oligo-nucleosomal DNA fragments in the "ladder" detected by gel electrophoresis. The scheme in (**B**) illustrates measurement of inter-nucleosome distances taking into account all nucleosome reads, including those originating from different DNA molecules.

Figure S3. Comparison of genome-wide nucleosome occupancy maps. (**A**) Density scatter plots for nucleosome occupancy per base pair in the CC set (y axis) *vs.* that in the 'In Vivo (YPD)' set (x axis) (29). The color of each region represents the number of data points mapped to that region. The Pearson correlation coefficient between the maps is indicated. (**B**) Same as in **A**, comparing the CC set with the 3AT set. Since the length information is not available for the YPD set, all the fragments in the CC and 3AT sets (with the length from 120 bp to 180 bp) are used for analysis.

Figure S4. Distance auto-correlation function for yeast (red) and nematode (blue) nucleosomes. The function was calculated using two approaches (see Methods). In our approach (**A, B**) multiple occurrences of nucleosomes in the same position were summed, while in the approach used by Valouev *et al.* (28) multiple occurrences of nucleosomes in the same position were counted only once (**C, D**). For the sake of comparison, the entire CC set (~16 million sequences) is used here, since nematode nucleosomes (~44 million) were obtained using a single-end sequencing technique (28) and thus, the precise lengths of the nucleosomal DNA fragments are unknown.

Figure S5. WW profiles in +1 nucleosomes for 3AT-induced genes: all 234 genes (**A**), the 45 most strongly induced (top-45) genes (**B**) and the 45 least induced (bottom-45) genes (**C**). The NCP sequences are 'oriented' based on the direction of transcription of the genes. The fragments are 'center-aligned' with 20-bp extension in both directions. Three base pair running averages are shown. The curves for the CC and 3AT sets are depicted in red and green respectively. The circles in (**A**, **B**) mark the peaks, separated by ~10 bp, in the linker DNA downstream of the core particle.

Figure S6. WW profiles in -1 and +2 nucleosomes for the 234 genes induced by 3AT: all 234 genes (**A, D**), the 45 most strongly induced (top-45) genes (**B, E**) and the 45 least induced (bottom-45) genes (**C, F**).

(**A-C**), -1 nucleosomes. The NCP sequences with midpoints located between coordinates -320 and -160 relative to the TSS of the gene are selected.

(**D-F**), +2 nucleosomes. The NCP sequences with the midpoints located between coordinates +141 and +305 relative to the TSS of the gene are selected.

Three base pair running averages are shown, symmetrized with respect to the dyad at base-pair step 73.5 (denoted by a dashed line). The NCP sequences are 'center-aligned' with 20-bp extensions in both directions. The curves for the CC and 3AT sets are depicted in red and green, respectively. The circles in (**B, E**) indicate a pronounced peak at approximately the same location as observed in Figure 7C (the bottom-45) but not in Figure 7B (the top-45 group of the 234 genes). In this regard, the -1 and +2 nucleosomes differ from the +1 nucleosomes (Figure 7).

Figure S7. Positioning of WW (AA:TT + AT +TA) dimers in various yeast nucleosome sets before and after realignment. (**A, B**) Combined frequencies of occurrence of WW dimers for a set containing 54,753 nucleosome positions published by Mavrich *et al.* (30). We used the dyad positions given in Table S1 of ref. 30 and extracted the 147-bp nucleosomal sequences with 20-bp linkers from the yeast genome. The resulting sequences were aligned about the dyad positions. (**C, D**) WW profiles for the CC set with L = 147-152 bp (16). Only 3-bp running averages are shown. The location of the dyad is indicated by a vertical dashed line. Note that the results presented in the main part of our manuscript are based on the nucleosomal sequences obtained without re-alignment (compare (**C**) with Figure 6). In (**D**), all the sequences were realigned using the sequence patterns critical for nucleosome positioning (44). Specifically, for a given sequence with the dyad at position X, 10 additional sequences with the dyads at positions X-5, X-4, …, X-1, X+1, …, X+4, X+5 were evaluated by the "Set 2" function (44). The sequence with the highest "Set 2" score was used for further analysis – that is, each nucleosomal sequence was shifted by no more than 5 bp. The red circles in (**B**, **D**) emphasize pronounced peaks at locations #68, #78 (close to the dyad). This is a consequence of re-alignment because in Figure 6 and Supplementary Figure S7C (the raw data) these peaks are practically negligible.

Figure S8. Nucleosome organization around the 5' end of the 45 most strongly induced (top-45) genes (**A**) and the 45 least induced (bottom-45) genes (**B**). The nucleosome occupancy profiles relative to TSS (position 0) are presented for 3AT set (green) and CC set (red). The occupancy value at each nucleotide is normalized by summing all the nucleosome sequences covering this nucleotide and dividing that number by the average number of nucleosome sequences per base pair across the genome.

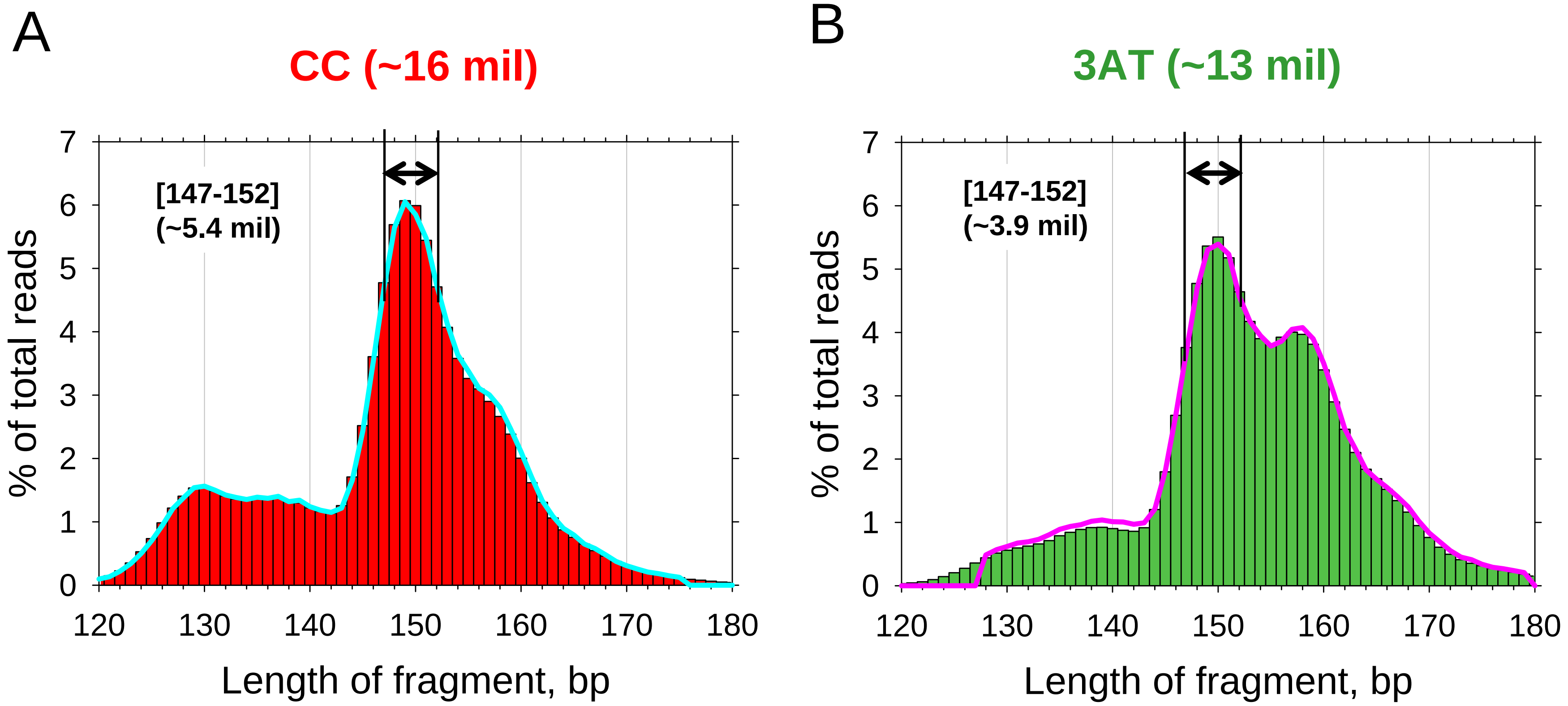
A
CC (~16 mil)
[147-152]
(~5.4 mil)
% of total reads
0
1
2
3
4
5
6
7
120
130
140
150
160
170
180
Length of fragment, bp
B
3AT (~13 mil)
[147-152]
(~3.9 mil)
% of total reads
0
1
2
3
4
5
6
7
120
130
140
150
160
170
180
Length of fragment, bp

Figure S1

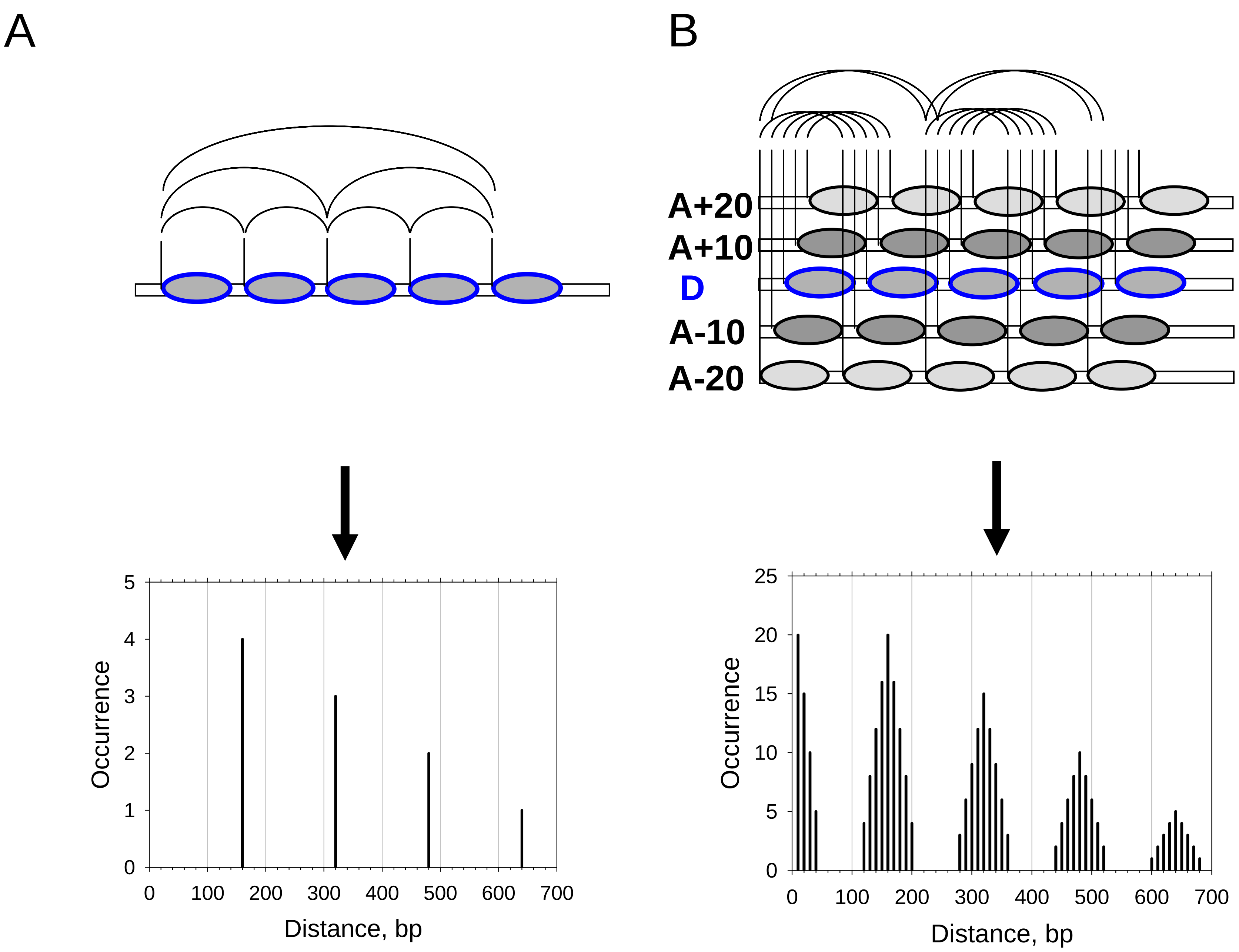

A
B
A+20
A+10
D
A-10
A-20
5
4
3
2
1
0
Occurrence
0 100 200 300 400 500 600 700
Distance, bp
25
20
15
10
5
0
Occurrence
0 100 200 300 400 500 600 700
Distance, bp

Figure S2

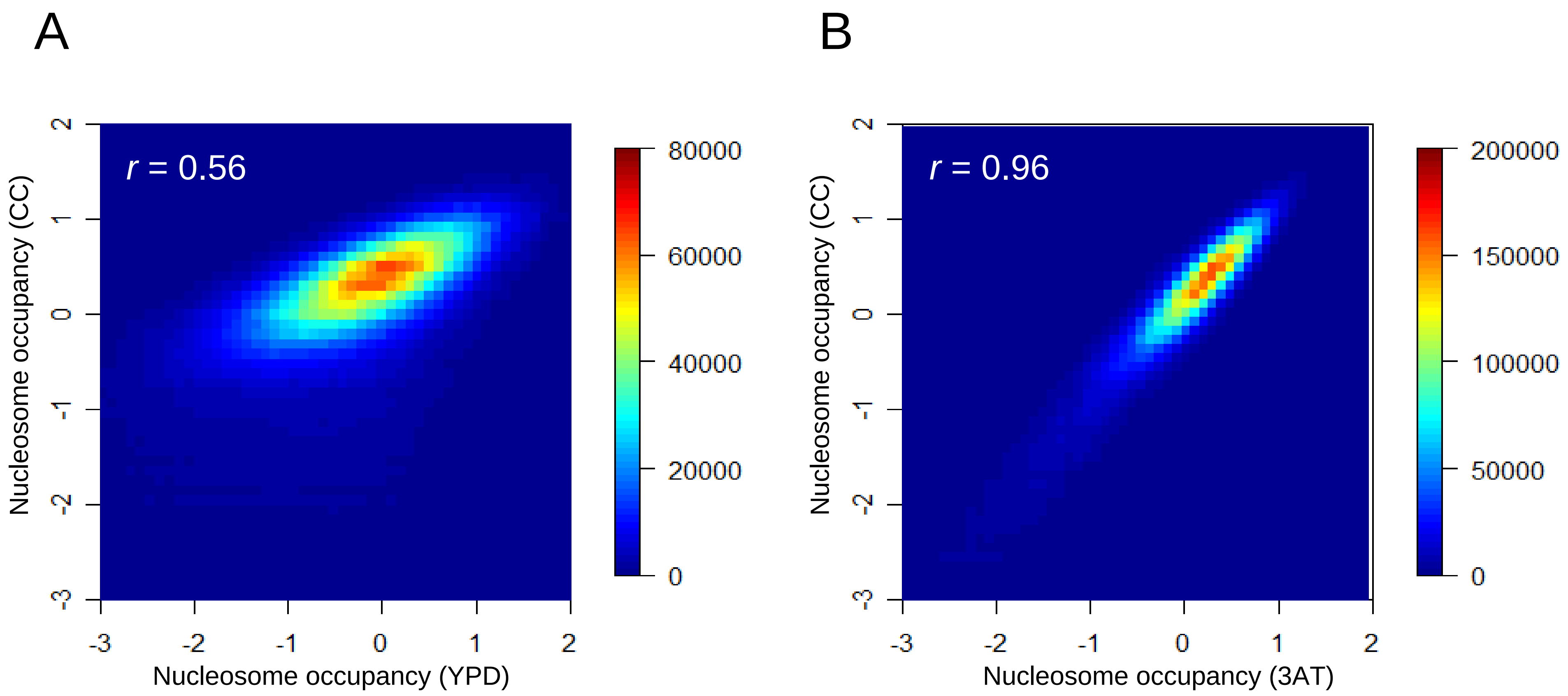
A
r = 0.56
Nucleosome occupancy (CC)
Nucleosome occupancy (YPD)
-3
-2
-1
0
1
2
80000
60000
40000
20000
0
B
r = 0.96
Nucleosome occupancy (CC)
Nucleosome occupancy (3AT)
200000
150000
100000
50000
0

Figure S3

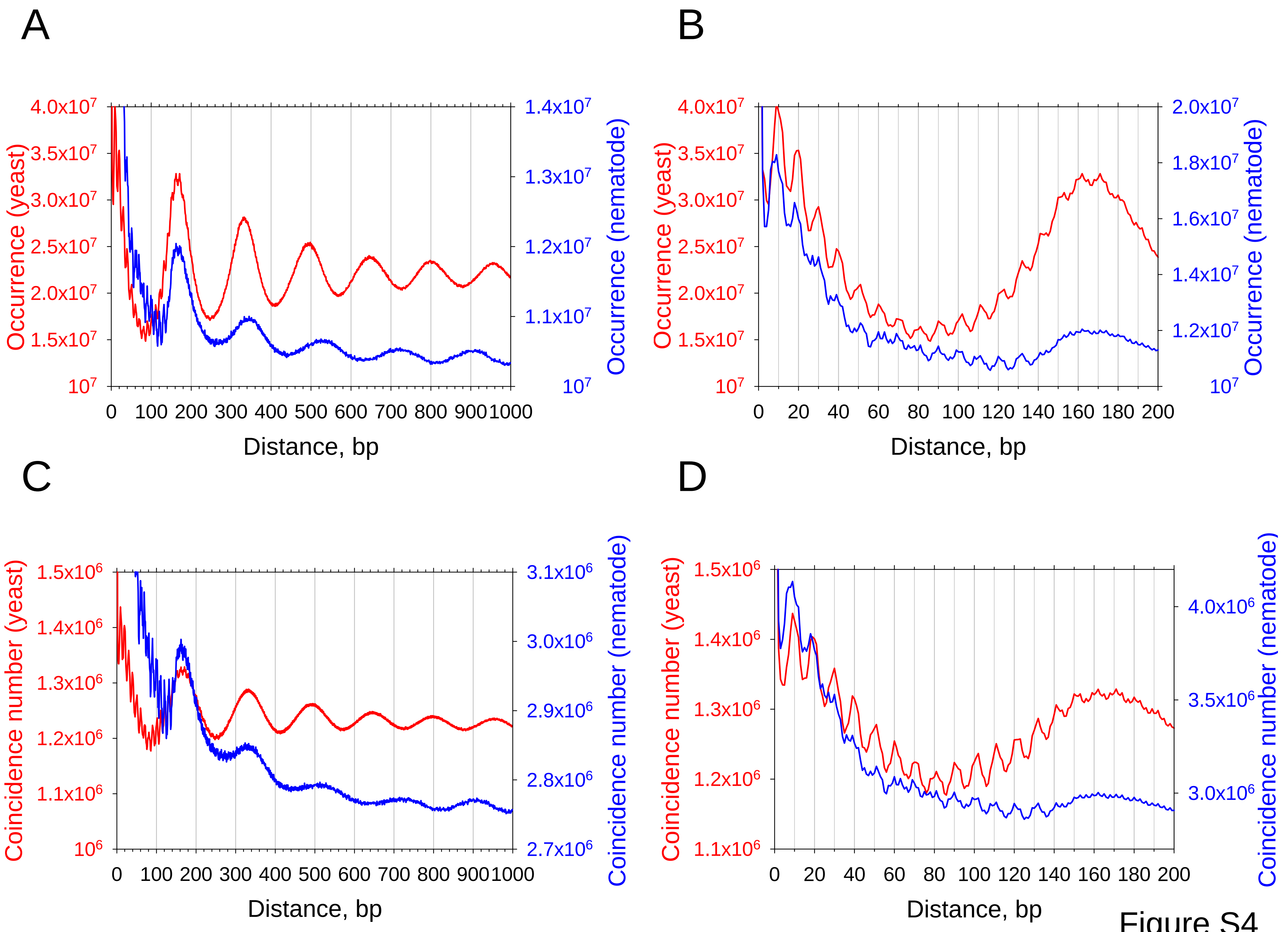
A
B
C
D
Occurrence (yeast)
Occurrence (nematode)
Coincidence number (yeast)
Coincidence number (nematode)
Distance, bp
4.0x10^7
3.5x10^7
3.0x10^7
2.5x10^7
2.0x10^7
1.5x10^7
10^7
1.4x10^7
1.3x10^7
1.2x10^7
1.1x10^7
2.0x10^7
1.8x10^7
1.6x10^7
1.4x10^7
1.2x10^7
1.5x10^6
1.4x10^6
1.3x10^6
1.2x10^6
1.1x10^6
10^6
3.1x10^6
3.0x10^6
2.9x10^6
2.8x10^6
2.7x10^6
4.0x10^6
3.5x10^6
3.0x10^6
0 100 200 300 400 500 600 700 800 900 1000
0 20 40 60 80 100 120 140 160 180 200

Figure S4

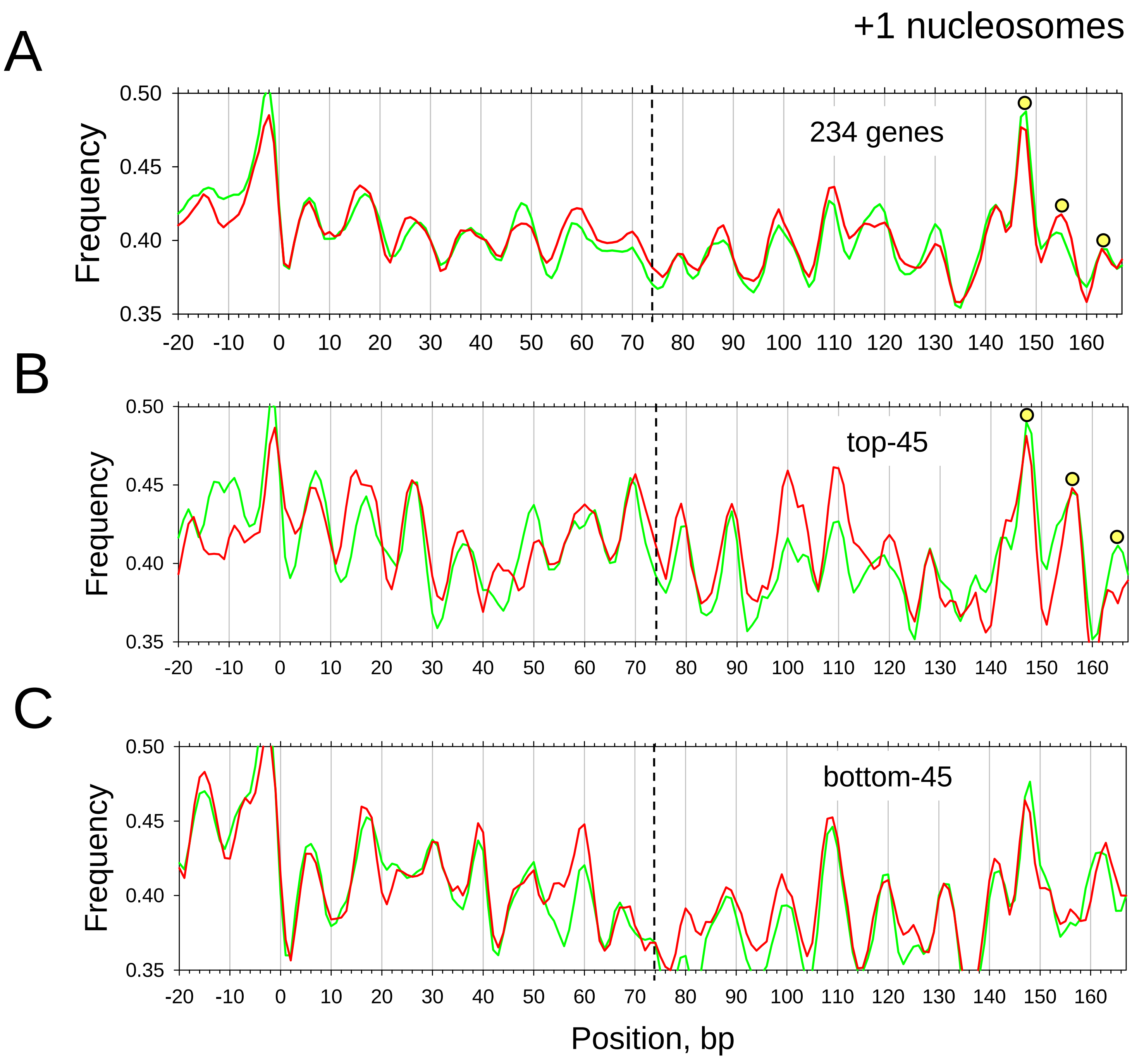

A
+1 nucleosomes
234 genes
B
top-45
C
bottom-45
Frequency
0.50
0.45
0.40
0.35
-20 -10 0 10 20 30 40 50 60 70 80 90 100 110 120 130 140 150 160
Position, bp

Figure S5

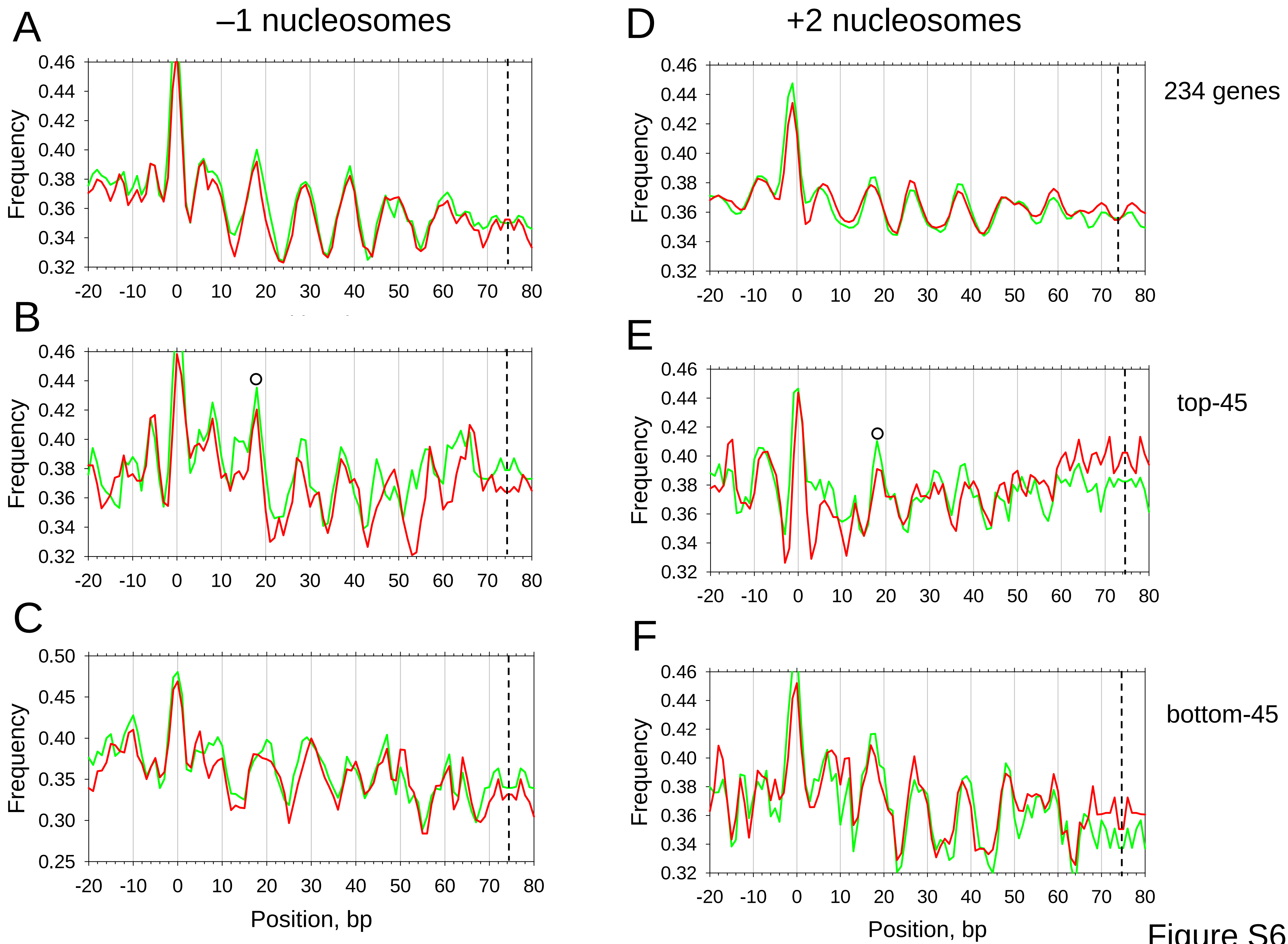
A
–1 nucleosomes
D
+2 nucleosomes
234 genes
B
E
top-45
C
F
bottom-45
Frequency
Position, bp
0.46
0.44
0.42
0.40
0.38
0.36
0.34
0.32
0.50
0.45
0.35
0.30
0.25
-20
-10
0
10
20
30
40
50
60
70
80

Figure S6

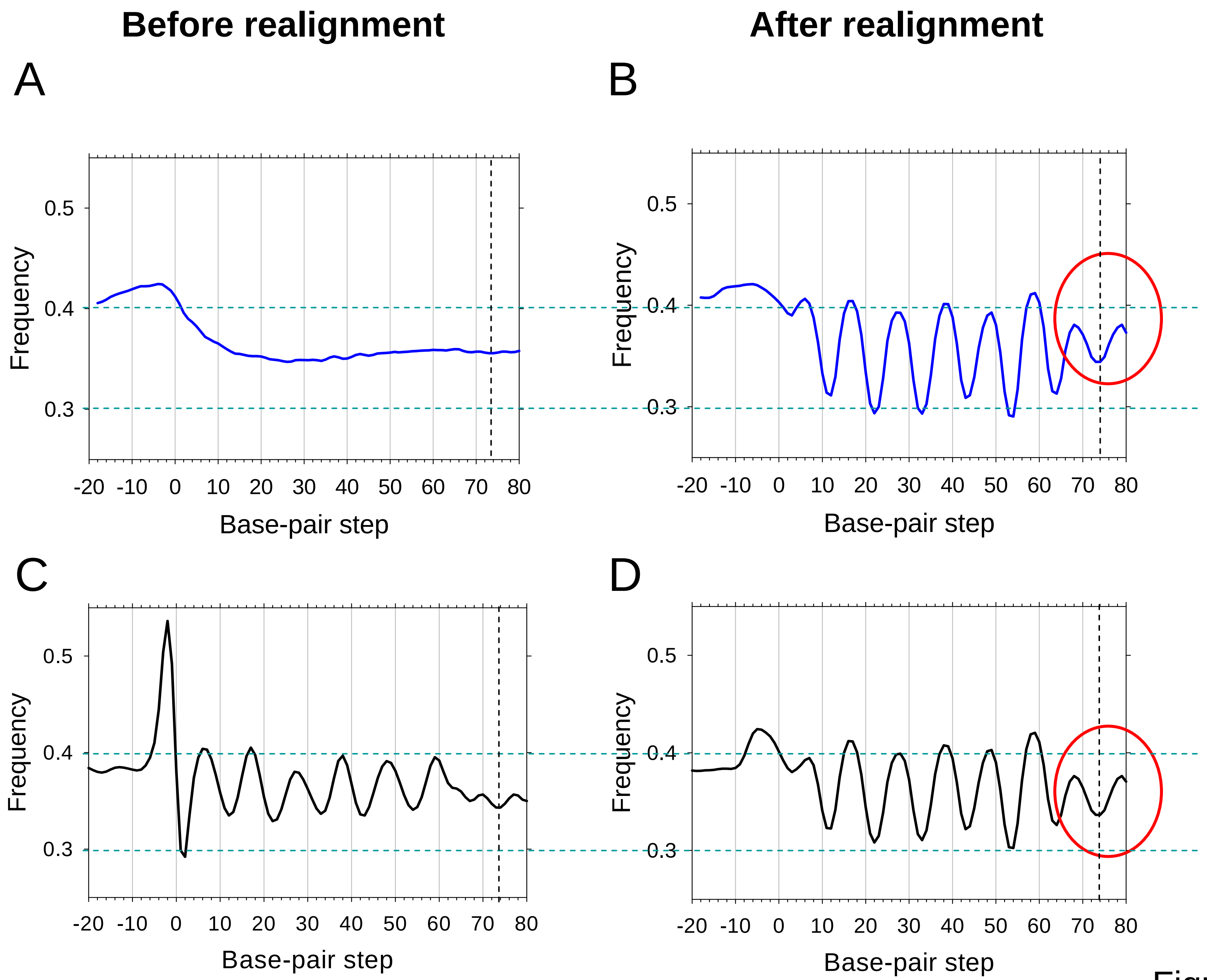
Before realignment
After realignment
A
B
C
D
Frequency
0.5
0.4
0.3
-20
-10
0
10
20
30
40
50
60
70
80
Base-pair step

Figure S7

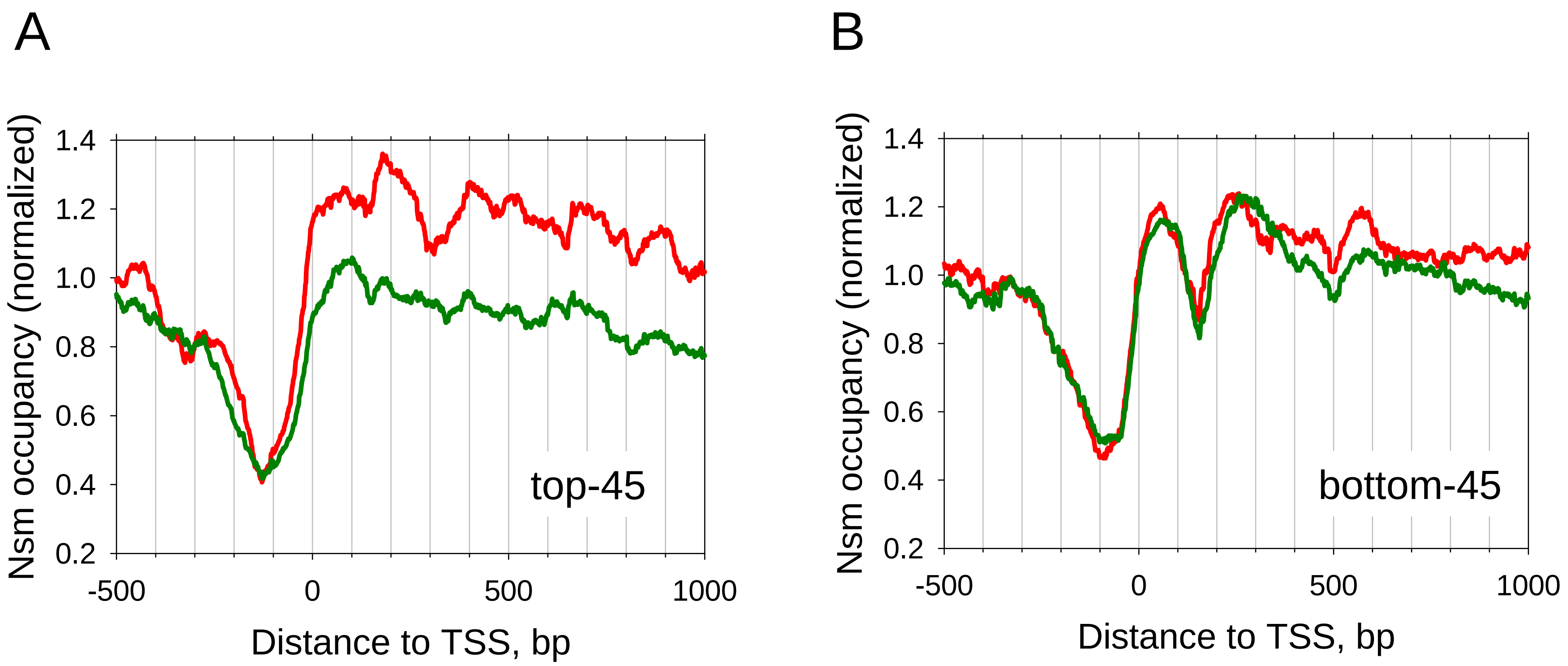
A
B
Nsm occupancy (normalized)
1.4
1.2
1.0
0.8
0.6
0.4
0.2
-500
0
500
1000
Distance to TSS, bp
top-45
bottom-45

Figure S8

Table S1. Base content (%) across the MNase cleavage sites at the ends of nucleosomal DNA fragments [a]

**Position**	**-2**	**-1**	**0**	**+1**	**+2**	**+3**
**A**	**31**	**36**	34	39	21	27
**T**	**33**	**34**	**49**	**54**	16	24
**G**	17	12	6	3	**42**	27
**C**	19	18	11	51	21	22
**W(A+T)**	**64**	**70**	**83**	**93**	37	51

[a] MNase cleavage occurs between positions 0 and +1.

The 147-152 bp long NCP fragments from the CC set were analyzed (5,368,041 in total). For each NCP fragment, both strands were aligned to their 5'-ends. The values shown in bold indicate that the MNase 'degenerate consensus' can be defined as WWT|TG, where vertical bar denotes the cleavage site (W stands for A or T).
